# Density and Biomass Estimates by Removal for an Amazonian Crocodilian, *Paleosuchus palpebrosus*

**DOI:** 10.1371/journal.pone.0156406

**Published:** 2016-05-25

**Authors:** Zilca Campos, William E. Magnusson

**Affiliations:** 1 Laboratório de Vida Selvagem, Embrapa Pantanal, Corumbá, Mato Grosso do Sul, Brazil; 2 Coordenação de Biodiversidade, Instituto Nacional de Pesquisas da Amazônia, Manaus, Amazonas, Brazil; University of South Carolina, UNITED STATES

## Abstract

Direct counts of crocodilians are rarely feasible and it is difficult to meet the assumptions of mark-recapture methods for most species in most habitats. Catch-out experiments are also usually not logistically or morally justifiable because it would be necessary to destroy the habitat in order to be confident that most individuals had been captured. We took advantage of the draining and filling of a large area of flooded forest during the building of the Santo Antônio dam on the Madeira River to obtain accurate estimates of the density and biomass of *Paleosuchus palpebrosus*. The density, 28.4 non-hatchling individuals per km^2^, is one of the highest reported for any crocodilian, except for species that are temporarily concentrated in small areas during dry-season drought. The biomass estimate of 63.15 kg*km^-2^ is higher than that for most or even all mammalian carnivores in tropical forest. *P*. *palpebrosus* may be one of the World´s most abundant crocodilians.

## Introduction

It is extremely difficult to obtain density estimates of most species of crocodilians because they inhabit inundated areas with difficult access. It is also usually difficult to meet the assumptions of mark-recapture techniques because crocodilians are wary and avoid recapture. Bayliss, 1987 [1] used capture and resighting techniques to estimate the density of large *Crocodylus porosus*, but most species cannot be as easily seen during the day. Also, animals less than a year old, referred to as hatchlings, are usually not included because of their high mortality rates and subsequent changes in density throughout the year. For these reasons, most descriptions of abundance of crocodilians are given as the number of nonhatchling crocodilians seen per kilometer of bank and no correction is made for individuals that are not seen.

Some species of crocodilians, such as *Caiman crocodilus* [2–5] and *Melanosuchus niger* [6,7] reach high observed densities during the dry season when waterbodies contract, but many individuals may remain in inaccessible places [8] and it is not known how these estimates relate to wet-season densities when individuals disperse through flooded habitats to feed.

Magnusson and Lima, 1991 [9], estimated 13 adult territory-holding *Paleosuchus trigonatus* in an area of 4.8 km^2^, giving a density of 2.7*km^-2^ for an area of rainforest in central Amazônia, but that study was based on careful mapping of home ranges and took 8 years to complete. It did not estimate densities of subadults. *Paleosuchus palpebrosus* is known to occur in flooded forests in central Amazonia [10–13], but there are no estimates of its density. In this study, we take advantage of the construction of the Santo Antônio hydro-electric dam on the Madeira River to estimate the density of *P*. *palpebrosus* in an area of flooded forest by complete catch of all individuals in a 5.6 km^2^ area.

## Material and Methods

The Santo Antônio hydro-electric dam is situated on the Madeira River, Rondônia State, Brazil [14] and its reservoir was filled in 2011. An area of flooded forest called the Engenho Velho immediately upstream of the dam had to be reclaimed as part of the dam construction (8°47´S; 63°56´O). The Engenho Velho forest covered 5.6 km^2^ and was the only flooded forest on the left bank of that stretch of river, which was dominated by fast-flowing rapids and relatively steep banks. Three streams, Igarapezinho, Peixes e Cachoeirinha, drained the forest.

The climate in the area is strongly seasonal, with most rain falling during the wet season (October to April). The mean annual temperature varies from 25°C and 27°C and mean annual rainfall varies of 1400 to 2000 mm [15].

Before the construction of the dam, the Engenho Velho forest was flooded by water from the Madeira River each year from December or January to March or April. During the rest of the year, water was largely confined to the streams. Vegetation consisted largely of small trees and large bushes with some areas covered by rushes and other herbaceous plants. The trees formed dense thickets, making foot travel in the dry season or boat travel in the wet season difficult and too slow to permit effective surveys of caimans.

In May of 2009 Santo Antônio Energia (SAE), the company responsible for construction of the dam, pushed up earthen walls around the dry watercourses leading into the streams to avoid caimans migrating into the area from the surrounding highlands, which are within the limits of the city of Porto Velho and unlikely to support many caimans in any case. All construction activities and the concentration of fauna-rescue teams in the area were planned to allow complete catch of caimans in the area.

**Fig 1:**
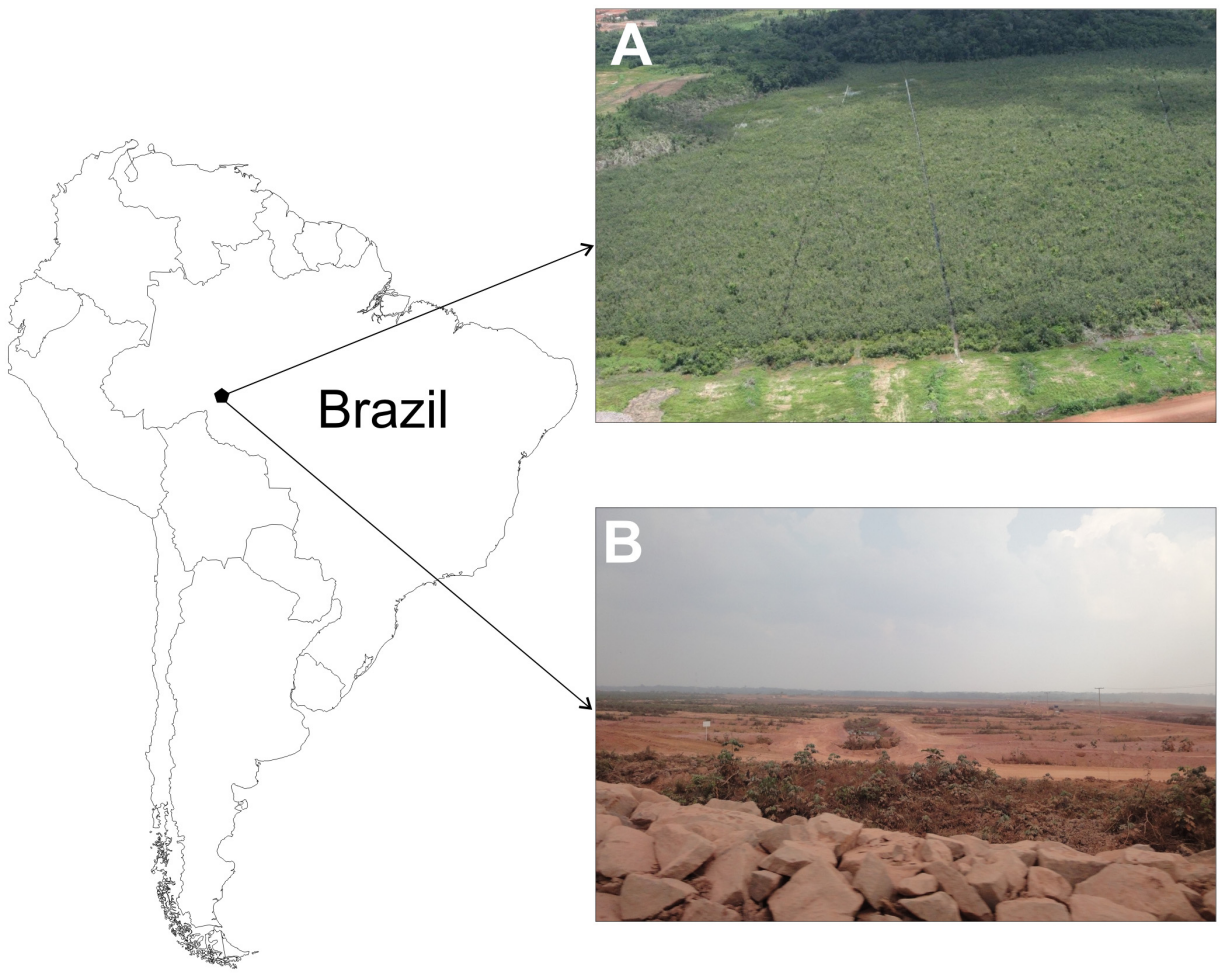
Photos of the Engenho Velho forest study area before (A) and after (B) installation of the Santo Antônio Hydro-electric Dam on the Madeira River, Rondônia, Brazil.

Before starting the landfill, SAE employees cut 12 trails that crisscrossed the Engenho Velho forest so that fauna-rescue teams could search the area for caimans (Fig 1A). Starting in May 2009, four to eight assistants searched the area at least three times a week from wooden canoes or by walking along the access trails, both during the day and night. The area received a total of 2,194 man hours of search throughout the study period. Surveys were concentrated in periods when new areas became accessible as the landfill and drainage progressed.

The forest was gradually removed, starting in July 2009 and ending in November 2010 (Fig 1B). The assistants accompanied the removal of the vegetation and the reclamation of the area with landfill, and captured any caimans that were unearthed.

Snout-vent length (from the tip of the snout to the posterior edge of the cloaca) was measured on all caimans captured with a measuring tape graduated in mm. The mass of the caimans was recorded with a 300g, 1 kg, 10 kg or 50 kg spring scales (limit of readings 0.1, 10 g, 100 g and 100 g, respectively), depending on the size of the caiman. Animals with SVL > 60 cm were marked with an aluminum tag placed in the webbing of the left hind foot and the remainder by cutting the tips of a unique combination of three protruding double and single tail scutes [16]. The sex of animals with SVL > 40 cm was determined by cloacal probing.

Most (138) captured animals were translocated by boat to Lago São Miguel (8° 35`S; 63° 48`O), which is 30 km upstream of the capture site. Ninety four individuals were released between Quilha (8° 44`S; 63° 56`O) and Lago Maravilha (8° 43`S; 63° 56`O), about five kilometers upstream of the Engenho Velho forest.

To determine whether caimans were dispersing in the river to or from the study area, one of us (ZC) carried out nocturnal surveys for caiman in the river from about 3 km downstream to 10 km upstream of the forest in October 2007, July and October 2008, and August 2010, using an outboard canoe and powerful spotlight.

### Research permits

The research project was approved by the Brazilian Environmental Agency (IBAMA permit N°. 017/02) and by the Chico Mendes Institute for Biodiversity Conservation (ICMBio permanent license N°.13048–1) for capture and marking caimans (relevant legislation IN N° 154/2007). All procedures followed ethical practices for animals following Decree Number 6899 of 15 July 2009, and were approved by the Committee on Animal Ethics of the Brazilian Agricultural Research Organization (EMBRAPA 009/2016). No collection of biological material (blood, tissue etc.) was used in this study. This species is classified by the International Union for Conservation of Nature (IUCN) as Lower Risk, least concern for conservation.

## Results

The *P*. *palpebrosus* individuals in the Engenho Velho forest were essentially isolated from other caimans of the same species. Only two *P*. *palpebrosus* were recorded within 10 km of the forest during the three years of surveys of the main river. That section of river with strong rapids appeared to be inhospitable to caimans and only one *Caiman crocodilus*, one *Melanosuchus niger* and two *P*. *trigonatus* were seen during the same surveys. Four individuals of *P*. *palpebrosus* translocated to Quilha were captured when they returned about 5 km to the river in front of the Engenho Velho forest area, but only after periods of 150, 150, 270 and 330 days.

The number of caimans captured varied during the study period due to changes in water level, access and the amount of forest remaining, so we could not construct a catch-out graph, but the intensity of searching and the fact that there was no habitat left at the end of the study leads us to believe that almost all caimans in the area were captured. A female with SVL = 48.5 cm that had been buried during landfill was found on 14 August 2010, so some caimans may have been buried where we could not find them.

Animals with SVL ≤ 22 cm represented 31.5% of captures. Based on previous studies[12,17], these animals would have been less than 12 months old and such individuals are regarded as hatchlings and not usually included in density estimates. A total of 159 non hatchling individuals were captured in the 5.6 km^2^ area, giving a density of 28.4*km^-2^.

**Fig 2:**
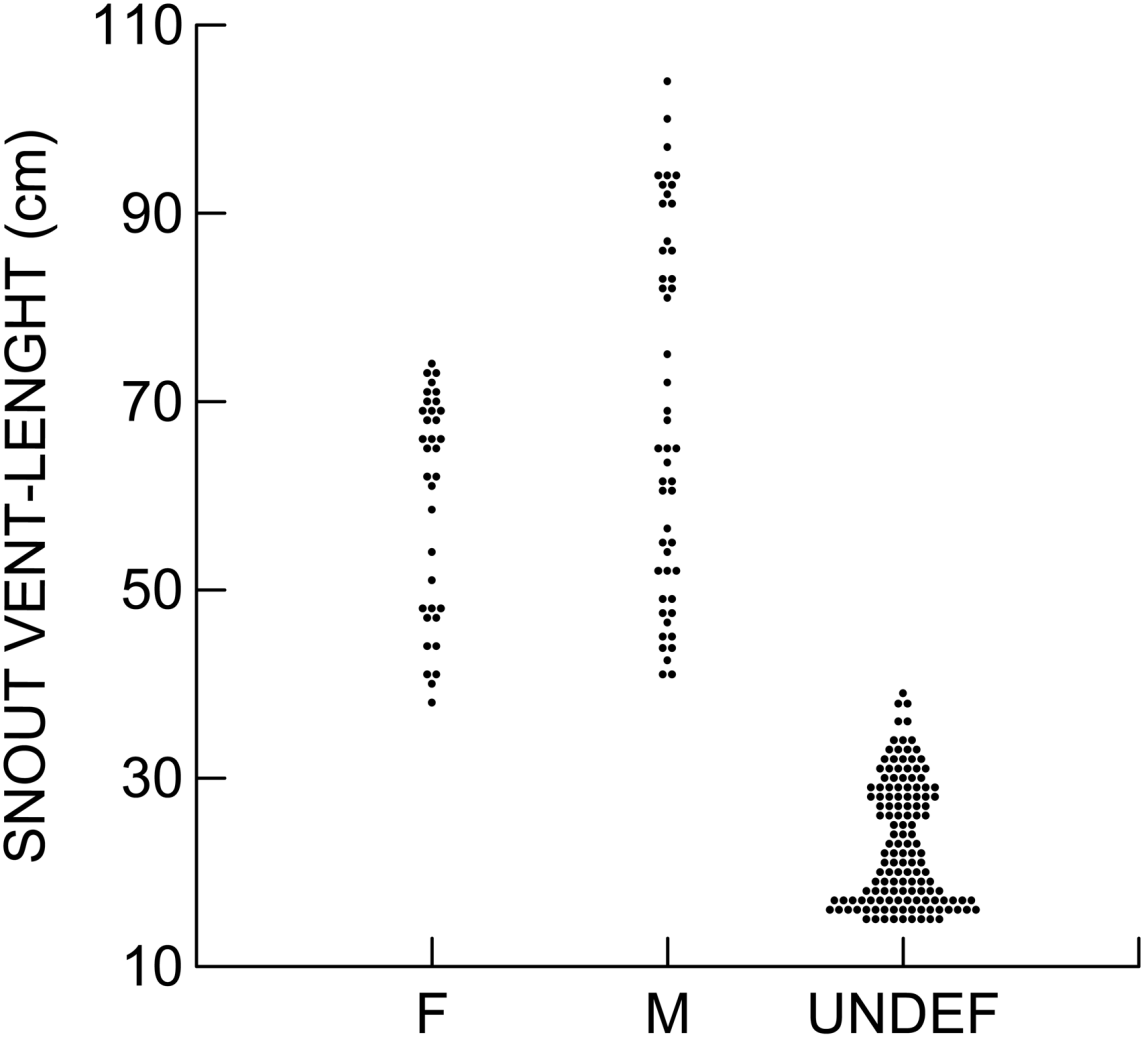
Size distributions of *Paleosuchus palpebrosus* in the Engenho Velho forest, Madeira River, Rondônia, Brazil. F = females; M = Males; Undef = Undefined.

The size distribution of dwarf caimans captured (Fig 2) indicates that the isolated individuals in the Engenho Velho forest represented a self-sustaining population with large numbers of hatchlings and subadults. The total biomass of *P*. *palpebrosus* was 353.63 kg, indicating a biomass density of 63.15 kg*km^-2^. We caught 9 males with SVL > 75 cm and 11 females with SVL > 65cm, with a combined mass of 228 kg. Assuming that these individuals were reproductively mature with fixed home ranges, this gives a biomass of adult animals of 40.7kg*km^-2^.

We compared the size distributions of dwarf caimans collected in intensive studies in a region near the Pantanal and an area of flooded forest near the Amazon River [11], both areas more than 600 km from the Santo Antônio dam, with that of the species in the Engenho Velho forest. In both cases, Kolmogorov-Smirnov tests indicated no significant differences for males (P = 0.563 and P = 0.687, respectively) or females (P = 0.483 and P = 0.109, respectively).

## Discussion

As the landward access to the flooded forest was isolated before surveys began, and transit through the main river so low, immigration during the study period would have had negligible effect on our density estimates. The Engenho Velho forest caimans were apparently essentially isolated from other caimans in the region and so probably represented a self-sustaining population.

Although estimates of relative density may be obtainable in some situations, estimation of absolute densities of crocodilians by conventional methods is not generally feasible and the complete destruction of a large area with the objective of collecting all caimans is generally not ethically or economically feasible. Therefore, the construction of the Santo Antônio hydro-electric dam offered an unusual opportunity to obtain accurate density estimates for a crocodilian.

We estimated a biomass of about 63 kg*km^-2^ for all nonhatchling *P*. *palpebrosus* in the Engenho Velho forest. However, it is difficult to compare the densities to those of most other crocodilians, for which only relative density in the form of individuals seen per kilometer of shoreline has been reported. Data on territory-holding *P*. *trigonatus* in central Amazonia [9] indicate densities of about 2.7 individuals*km^-2^. Based on studies near the Amazon River and in regions near the Pantanal [11], we assume that *P*. *palpebrosus* reproduces and has fixed home ranges from SVL = 75.0 cm for males and SVL = 65.0 cm for females. Dividing the number of adults present by the area of the forest indicates that there would have been about 6.6 territory-holding individuals*km^-2^ in the Engenho Velho forest.

The biomass density for those large individuals would have been about 40.7 kg*km^-2^, which is within the range (34.4–59.6 kg*km^-2^) estimated for *P*. *trigonatus* with fixed home ranges reported by Magnusson and Lima, 1991, and indicates that, like *P*. *trigonatus*, *P*. *palpebrosus* has higher biomass than that reported for a mammalian predator of similar size (6 kg*km^-2^) [18], all felids (8 kg*km^-2^) [19] or even all mammalian predators together in other tropical forests [20,21].

Although densities of *P*. *palpebrosus* appear to have been underestimated in the past, the size distributions of individuals captured opportunistically in limited areas were similar to those obtained in the total catch out in the Engenho Velho forest. Therefore, it is likely that size distributions from intensive studies can be used to describe population size distributions of *P*. *palpebrosus* even when most individuals have not been captured.

*Paleosuchus palpebrosus* is often considered rare and it is rarely the most commonly encountered crocodilian anywhere in its range [22, 23]. However, this may just reflect the difficulty of surveying its habitats. About 30% of the seven million square miles that make up the Amazon basin comply with international criteria for wetlands [24] and much of that is prime habitat for *P*. *palpebrosus*, so *P*. *palpebrosus* may be one of the highest biomass predators in the Amazon. However, the threats to wetland habitats of this species are intense in other regions of Brazil [25, 26].

## Supporting Information

S1 Appendix Locations of the releases sites, Santo Antônio Dam, Engenho Velho forest, and Porto Velho city indicated by black circles. White dotted line indicate nocturnal transect in the Madeira River, Rondônia, Brazil.
(TIF)
