# Supplementary figures and images for: Density and Biomass Estimates by Removal for an Amazonian Crocodilian, Paleosuchus palpebrosus
Source: PLoS One. 2016 May 25;11(5):e0156406. doi: 10.1371/journal.pone.0156406 (PMC4880199; doi:10.1371/journal.pone.0156406)

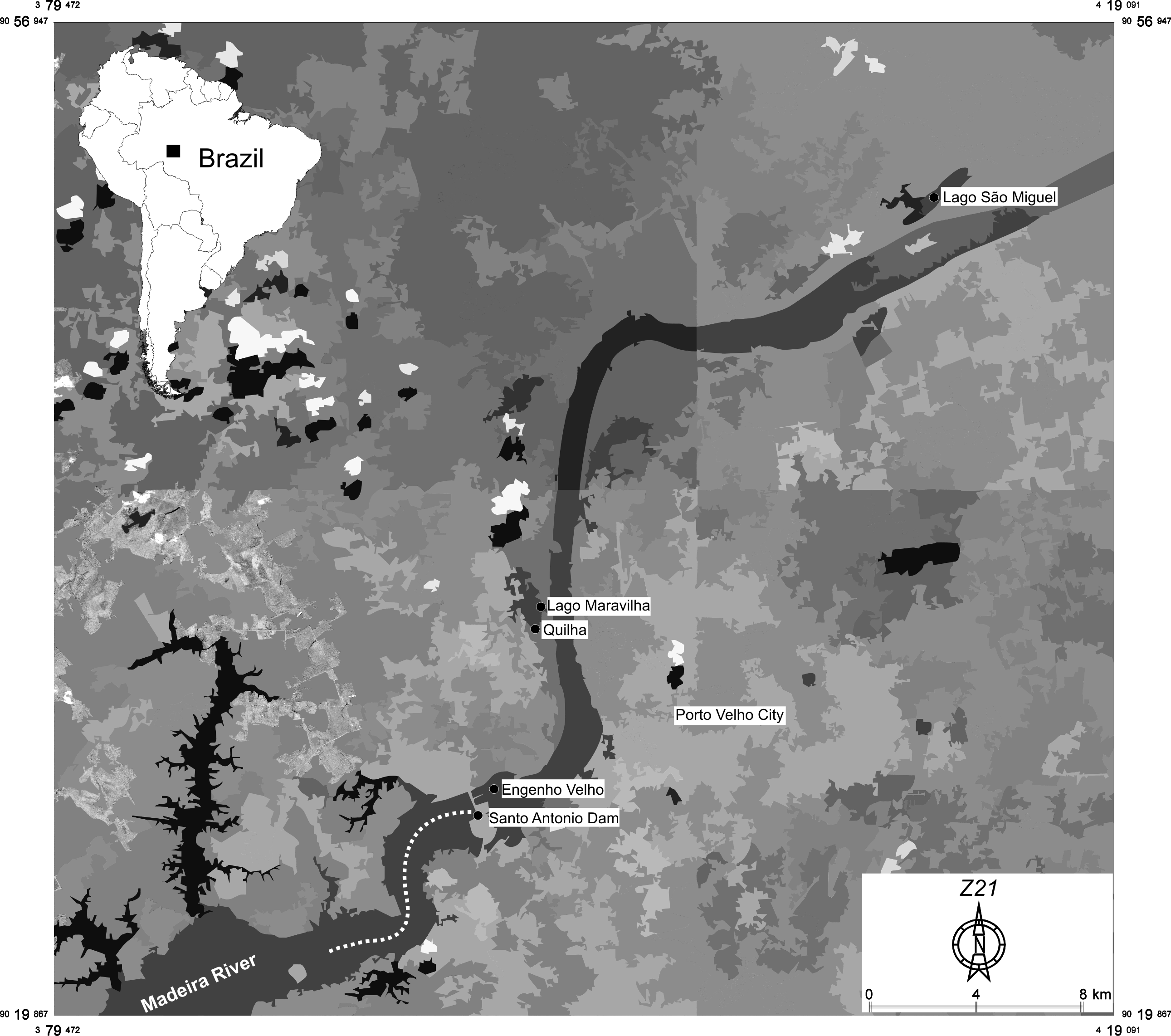

Supplement: S1 Appendix — White dotted line indicate nocturnal transect in the Madeira River, Rondônia, Brazil. (TIF) [file pone.0156406.s001.tif]
